# The School Malaise Trap Program: Coupling educational outreach with scientific discovery

**DOI:** 10.1371/journal.pbio.2001829

**Published:** 2017-04-24

**Authors:** Dirk Steinke, Vanessa Breton, Emily Berzitis, Paul D. N. Hebert

**Affiliations:** Centre for Biodiversity Genomics, University of Guelph, Guelph, Ontario, Canada

## Abstract

The School Malaise Trap Program (SMTP) provides a technologically sophisticated and scientifically relevant educational experience that exposes students to the diversity of life, enhancing their understanding of biodiversity while promoting environmental stewardship. Since 2013, the SMTP has allowed 15,000 students at 350 primary and secondary schools to explore insect diversity in Canadian schoolyards. Students at each school collected hundreds of insects for an analysis of DNA sequence variation that enabled their rapid identification to a species. Through this hands-on approach, they participated in a learning exercise that conveys a real sense of scientific discovery. As well, the students contributed valuable data to the largest biodiversity genomics initiative ever undertaken: the International Barcode of Life project. To date, the SMTP has sequenced over 80,000 insect specimens, which includes representatives of 7,990 different species, nearly a tenth of the Canadian fauna. Both surprisingly and importantly, the collections generated the first DNA barcode records for 1,288 Canadian species.

## Motivation

Primary and secondary school students spend much of their lives interacting with their school’s environment. According to an estimate from the Organisation for Economic Co-operation and Development, North American students spend about 8,000 hours within public academic institutions between the ages of 7 and 14 years [1]. Given such extensive exposure, they soon become deeply familiar with their physical environment, but they often have little knowledge of its place in nature. One of the primary goals of science is to understand our world. In order to advance students’ understanding of nature, it is critical that they be provided with learning experiences that foster the development of this knowledge [2]. First and foremost, educational activities must be participatory so that students are active agents in their learning [3].

Ideally, students should engage in activities that allow them to develop knowledge and comprehension by engaging in the two major components of scientific inquiry—experimentation and research [2]. They then need to apply these methodologies to advance their understanding of the world in order to develop the skills necessary to take knowledge beyond the classroom. Teachers are increasingly encouraged to offer their students the chance to explore nature from a scientist’s perspective [4], but they often experience difficulty in implementation. In an attempt to better support teachers, some universities have launched outreach programs aimed at improving both science education for students and the approaches employed for environment, science, technology, engineering, and mathematics (E-STEM) in the classroom [5–9]. Such outreach programs have demonstrated very positive effects on student learning and comprehension [10–12].

## DNA barcoding

DNA barcoding employs sequence diversity in a short gene fragment from a standardized position in the genome as a tool for species identification [13]. It is now widely employed in practical contexts ranging from monitoring pests [14] and species at risk [15] to supporting the detection of invasive species [16] and suppressing food fraud [17] and deceptive herbal products [18]. Reflecting a major international effort to build a reference library of DNA barcodes for all known species, records for nearly 600,000 species are now available in the Barcode of Life Data Systems (BOLD) [19]. Aside from its practical and scientific value, DNA barcoding provides an outstanding basis for science instruction because it bridges disciplines—e.g., ecology, genetics, and informatics. In addition, its workflows are simple enough that students can participate in all aspects of the analytical chain from specimen collection to data interpretation [5].

From its base in California, Coastal Marine Biolabs was the first organization to introduce DNA barcoding into an educational context through an interdisciplinary program entitled Barcoding Life’s Matrix [20]. Now national in scope, this project allows senior secondary school students (grades 11 and 12) and their teachers to extend parameterization of the global DNA barcode reference library. DNA barcoding has also been adopted in other educational contexts [21–23], and its power was signaled when it won the *Science* Prize for Inquiry-Based Science Instruction [24].

## The School Malaise Trap Program (SMTP)

The SMTP empowers students as citizen scientists by asking a very basic yet fundamental question: “Do you know what lives in your schoolyard?” By asking this question, the program aims to trigger a student’s intrinsic curiosity for her/his surroundings, ultimately fostering bioliteracy and environmental stewardship by generating the knowledge and skills to effect long-term environmental change.

**Fig 1:**
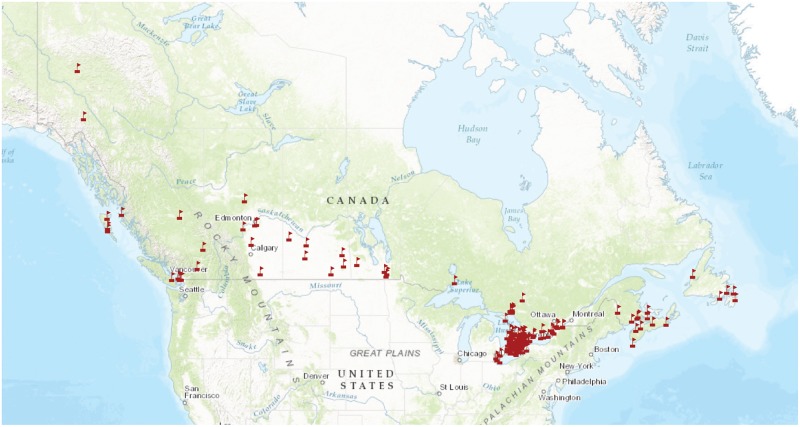
School Malaise Trap sampling sites from 2013–2015.

Since 2013, the SMTP has teamed up with students and educators in grades 4–12 (age 9–18 years) at schools across Canada (Fig 1) to explore the insect diversity in their schoolyards. The program employs Malaise traps, tent-like structures [25], along with DNA barcoding to quantify insect diversity. During the program, students are introduced to multiple E-STEM disciplines while running a sampling program and exploring DNA barcoding through hands-on, inquiry-based scientific research.

**Fig 2:**
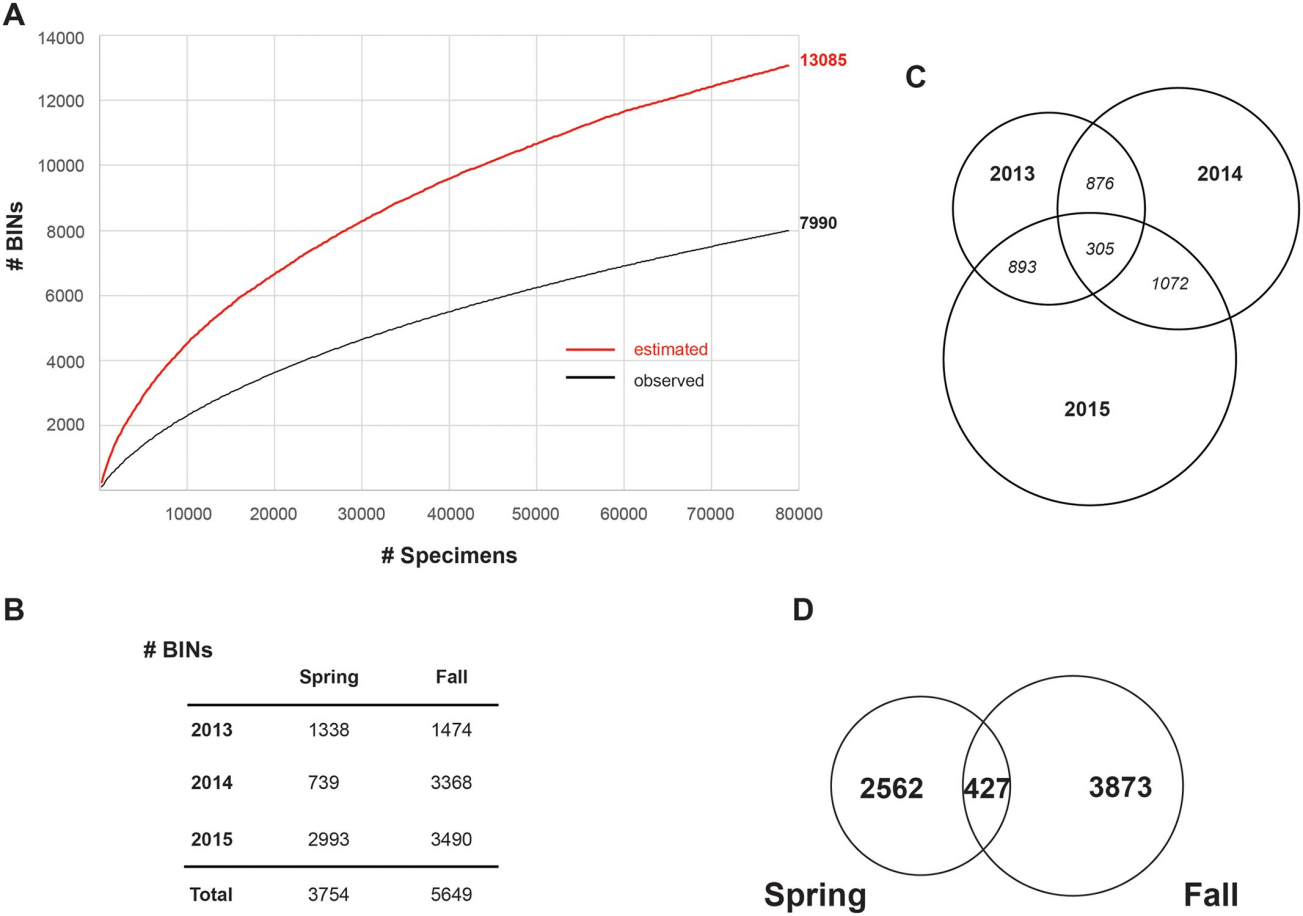
Barcode Index Number (BIN)/species counts across Canada. (A) BIN accumulation curve for all Malaise trap samples collected, depicting the presence of 7,990 species and indicating a total of 13,085 if sampling were complete. (B) BIN/species counts per year and season. (C) Venn diagram depicting BIN/species overlap between years. (D) Venn diagram of seasonal species overlap over 3 years.

The SMTP runs for 2 weeks of each spring and fall semester. All participating schools receive a package that includes grade-appropriate curriculum materials coupled with a Malaise trap and the other supplies needed to collect insects in their schoolyard. The resulting specimens are dispatched to the Centre for Biodiversity Genomics (CBG), where about 300 specimens from each school are barcoded [26–28]. Each class subsequently receives a program summary, a report on the performance of its trap, a species list, and an image library for the species collected at the school. Educators and students not only have the opportunity to analyze their school’s findings but can make comparisons with those from schools across Canada. These results allow students to assess trends in species abundance and biodiversity loss and make it possible for them to quantify diversity over time. Furthermore, by comparing rural sites with urban sites and by considering the habitats around each school, students can assess the impacts of urbanization on local biodiversity. Finally, the SMTP introduces teachers to a range of E-STEM tools that can significantly extend and enrich their instructional strategies—most notably, the online DNA barcode reference library, BOLD [19]. For example, it allows educators and students to explore data from past and present programs, to create species image libraries, to generate phylogenetic trees, and to view accumulation curves of species as an indication of sampling efficiency (e.g., Fig 2).

## Pedagogy

After receiving the SMTP package, students select a site where they deploy their Malaise trap. Once it is operational, they make daily recordings of temperature, weather conditions, and trap catch. The materials contain comprehensive lesson plans that address specific expectations across elementary and secondary curricula as well as supplementary cross-curricular activities and lesson extensions (see supporting information). Students are asked to formulate questions, hypotheses, and predictions regarding the relationships between the catch volume, weather, and surrounding habitat. Further investigations related to these questions reinforce the students’ ability to research, analyze, and justify their conclusions while utilizing a variety of formats (linguistic, numeric, symbolic, and media) to communicate ideas and results, all of which are crucial elements in the Partnership for 21st Century Skills framework (http://www.p21.org/framework).

While managing the Malaise trap over the 2-week sampling interval, students are encouraged to work collaboratively and to take ownership and responsibility for their research project. As a starting point for civic engagement, they receive informative and visually engaging material (see supporting information) that they take home to teach their families about the importance of biodiversity. Students and teachers are also encouraged to blog about their experience through the program website (http://malaiseprogram.com/), enabling them to interact with schools across the country. Every class successfully collected specimens, and half of them visited the SMTP site, with more than 90% contributing blog posts over the 2-week run. Blog entries ranged from simple statements relating to the location of their trap or weather conditions to detailed descriptions of extended lesson plans and activities that enriched the SMTP experience.

The SMTP emphasizes critical thinking skills in several core curriculum subjects, including biology, environmental science, mathematics, English, technology, and geography. Comprehensive lesson plans that address key expectations across elementary and secondary curricula are provided along with supplementary cross-curricular activities and lesson extensions. This program additionally immerses students in the subjects of environmental literacy, global awareness, and citizenship, with the goal of enhancing their commitment to environmental stewardship.

## Participation and student involvement

Since its activation in 2013, the SMTP has involved over 300 schools, 450 classrooms, and 15,000 students across nine Canadian provinces and two of its three territories. Demand for the program has consistently exceeded capacity, with an average of 130 schools competing for the 60 spots in each offering. This uptake is remarkable, as “marketing” has consisted of a single email announcement to principals and science department heads a few months in advance of the program start.

In order to better understand the engagement of students and teachers and how well the program met their expectations, an online survey was conducted after each offering, with response rates ranging from 30%–43%. Classroom teachers indicated strong satisfaction with the program (88%), adequacy of the materials (87%), and the program’s fit to their provincial curriculum (87%). A large majority (95%) stated that they would highly recommend the program to their peers. Furthermore, 91% observed that their students were strongly engaged in the program and demonstrated a positive response to this approach to learning. They emphasized that students quickly took ownership of the project and shared outcomes with their school and community, ultimately fostering accountability, which helped to ensure its success. According to one respondent, “students found the program enjoyable and an eye opener as to what is around them. Some were fascinated to know that species they had read about online were in their Malaise trap.” Another respondent noted that their “students saw the value in the program because it was real research, not just an example of how scientists collect information.” The knowledge that their school had contributed valuable data to a global research endeavor as well as their ability to access this information online was viewed as a particular highlight by educators and students. Many teachers expressed the view that “receiving information as to the number of total species and new species that were caught within our school community was enlightening.”

Because the SMTP aims to give students of all backgrounds the opportunity to participate, innercity schools in low-income areas and those with at-risk youth are given priority. A teacher from a school in Toronto wrote, “It was an important experience for our students and our community. Our school is part of an at-risk suburban community and I appreciate the opportunity to have my students involved in ‘real world science’ that takes them beyond the boundaries of their daily lives.” Schools in First Nations communities across Canada have also participated. Moreover, by partnering with conservation authorities and outdoor education centers, the SMTP extended its reach beyond classrooms, providing a broader audience with the opportunity to become acquainted with a new approach to surveying biodiversity.

## Scientific results

Aside from its educational impacts, the SMTP is contributing valuable scientific data. Past collections have included more than 300,000 insects and other arthropods, and 80,000 of them have been sequenced, revealing the presence of 7,990 species (Fig 2A and 2B). Within just 3 years, SMTP participants have collected nearly a tenth of the estimated 94,000 insect species that occur in Canada [29], which is a remarkable number since sampling is restricted to 2-week periods in the spring and fall, mostly in urban environments. Analysis suggests that more species await collection, as a Chao 1 [30] estimate for the total number of species present is 13,085 (Fig 2A). On average, each weekly sample included 525 specimens, but catches ranged from just 18 made by a school in Newfoundland in a chilly spring to a high of 22,500 by a school positioned near Lake Ontario. The number of species represented in the collections from each school varied from 6 to 186. Overlap between years was low (Fig 2C), reflecting modest sample sizes and the shift in sampling location, as priority is given to schools that are new to the program. Despite the limited number of sampling sites and the short duration of sampling, the SMTP added 1,288 new species records to the DNA barcode reference library for Canada. The fact that collecting activity for the SMTP occurs in early spring and fall, at times outside most academic research, may explain the high proportion (15%) of new barcode records. More species were collected in the fall than spring (3,873 versus 2,562), and just 427 species occurred in both seasons (Fig 2D). All DNA barcodes and their associated metadata (images, georeferences, and taxonomy) are publicly available, and several publications have already included data generated by the SMTP [29,31–35].

The SMTP represents an unprecedented biodiversity surveillance network driven by citizen scientists. Most species surveillance networks are geographically restricted and focus on small assemblages of species. Although some citizen scientist programs generate large volumes of data, most records are strictly observational [36]. By contrast, the SMTP data provide comprehensive insights into the biodiversity of a particular location, and each record is connected to a voucher specimen, allowing subsequent scientific study. By targeting classrooms, the SMTP combines rigorous data collection with science education for Canada’s youth.

## Conclusion

Our planet is facing an extreme loss of biodiversity; some estimates suggest that up to 100,000 species become extinct each year [37]. Given this fact, biodiversity loss is a critical environmental issue, one that cannot be ignored by present or future generations. The SMTP aims to address this environmental problem by inspiring Canada’s youth to pay attention to and, most importantly, to appreciate the life that surrounds them (even in their schoolyards!).

Given the simplicity of its design, the SMTP could be implemented in schools around the planet if the current cost (C$1,000 per school) for its delivery could be reduced. In fact, this barrier to adoption will soon be broken; new DNA sequencing platforms are enabling a major reduction in analytical costs. Consider a future in which student scientists around the world are responsible for leading the largest biomonitoring program ever undertaken, one tracking global trajectories in biodiversity from one generation to the next.

## Supporting information

S1 Table Spreadsheet for data entry. For weather, catchment tracking.
(PDF)

S1 Text Trap deployment instruction. (PDF)

S2 Text Program instruction for elementary classrooms. (PDF)

S3 Text Program instruction for secondary classrooms. (PDF)

S4 Text Flyer for students and for distribution to interested public. (PDF)

S5 Text Information brochure on program. (PDF)

S1 Document Collection Activity elementary classrooms. (ZIP)

S2 Document Collection Activity secondary classrooms. (ZIP)

S3 Document Collection Activity secondary classrooms. (ZIP)

S1 Video Trap build—Instructional video. (MP4)

## Abbreviations

BIN: Barcode Index Number
BOLD: Barcode of Life Data System
CBG: Centre for Biodiversity Genomics
E-STEM: environment, science, technology, engineering, and mathematics
SMTP: School Malaise Trap Program
