# Supplementary material for: The School Malaise Trap Program: Coupling educational outreach with scientific discovery
Source: PLoS Biol. 2017 Apr 24;15(4):e2001829. doi: 10.1371/journal.pbio.2001829 (PMC5402927; doi:10.1371/journal.pbio.2001829)
Supplement: S1 Table — For weather, catchment tracking. (PDF) [file pbio.2001829.s001.pdf]

School Malaise trap Program

Collection Records Spreadsheet

Teachers should keep a record of scientifically significant events (i.e. date, weather information, catch volumes observed, and incidence of trap disturbance) on the spreadsheet provided here. This record should be submitted along with the specimen collection bottles and the trap at the end of the collection period. These records sheets may be hung up in the classroom as a tool for student engagement throughout the deployment period.

EXAMPLE:

	Date	High Temp. (°C)	Cloud Cover (%)	Catch Volume	Notes (eg. trap disturbance)
**Week 1**	Monday, September 19	19°C	Partly cloudy (50%)	Below line 1 (~20 insects)*	Trap collapsed at back, had to re-secure. Specimen collection bottle appears fine.

**Indicate rough count of specimens only if the volume falls below the first tick line*

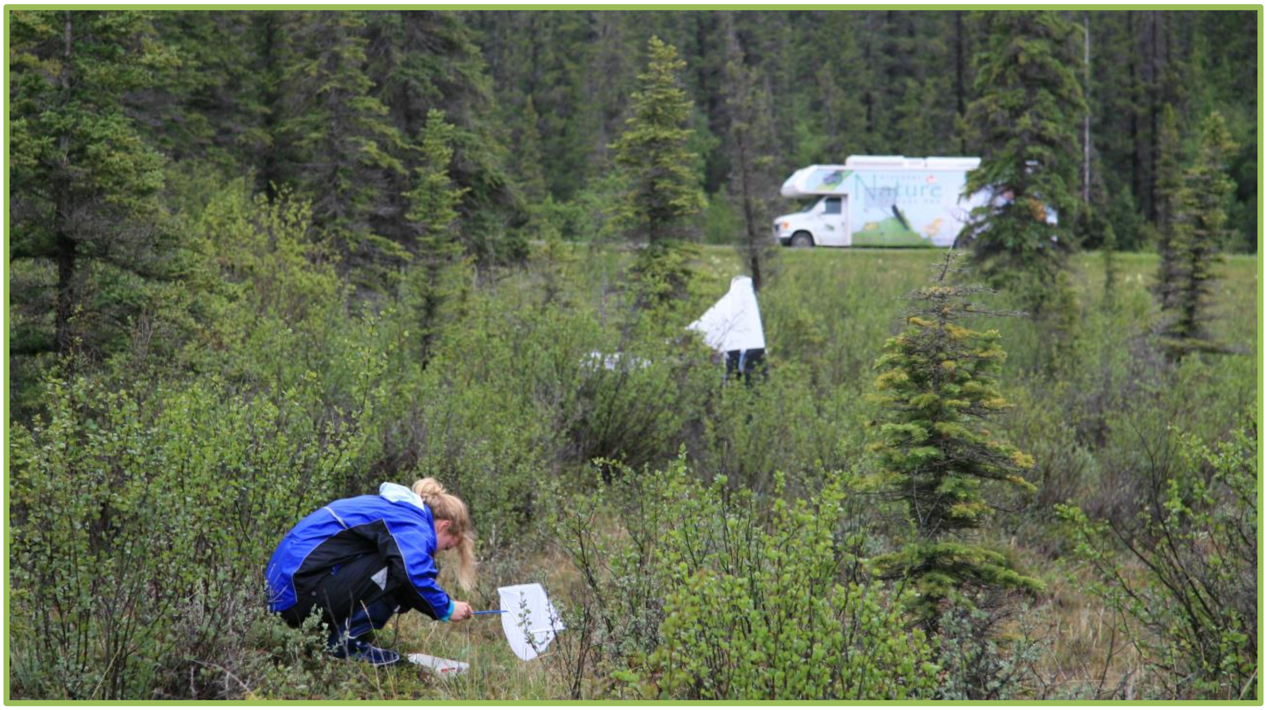

Collection Records Spreadsheet

Trap # (i.e. EQP-CLL#):

School: Teacher:

	Date	Daily High Temp. (°C)	Cloud Cover (%)	Catch Volume	Notes (e.g. trap disturbance)
**Week 1 (September 19 – 23, 2016)**	Monday, September 19				
	Tuesday, September 20				
	Wednesday, September 21				
	Thursday, September 22				
	Friday, September 23				
**Week 2 (September 26 – 30, 2016)**	Monday, September 26				
	Tuesday, September 27				
	Wednesday, September 28				
	Thursday, September 29				
	Friday, September 30				
