# Supplementary material for: The School Malaise Trap Program: Coupling educational outreach with scientific discovery
Source: PLoS Biol. 2017 Apr 24;15(4):e2001829. doi: 10.1371/journal.pbio.2001829 (PMC5402927; doi:10.1371/journal.pbio.2001829)
Supplement: S1 Text — (PDF) [file pbio.2001829.s002.pdf]

Malaise Trap Deployment

These standardized procedures must be followed to ensure consistency across all test sites for accurate data comparison, as well as to ensure that individual collections are successful. Please review this information carefully.

Malaise Trap Check List

The following materials will be provided in your program package. All items, with the exception of this SOP document and DVD, must be returned to the Centre for Biodiversity Genomics (CBG) at the Biodiversity Institute of Ontario (BIO) at the conclusion of the program.

- ☐ Malaise Trap Standard Operating Procedures (this document)
- ☐ Malaise trap EQL-CLL#_______________
  - ☐ Instruction sheet
  - ☐ 1 long pole
  - ☐ 1 short pole
  - ☐ 2 pre-labeled Nalgene collection bottles
  - ☐ 7 guy ropes
  - ☐ 14 tent pegs
- ☐ Malaise Trap Setup Instructional Video and program information (DVD)
- ☐ Trap Sign to be hung on the Malaise trap:
  - ☐ School Malaise Trap Program : Research in Progress sign
- ☐ Collection Period Recording Spreadsheet

Deployment

- One trap will be deployed at each school. The trap should be placed in a secure location, such as a fenced off area or somewhere that is not visible to the public. If this will pose a challenge or there are concerns regarding vandalism, you may consider an alternate location for the trap (e.g. teacher's private property).
- Each trap will be accompanied by a School Malaise Trap Program: Research in Progress warning sign to discourage disturbance. This will need to be attached to the front of the Malaise trap once it has been put into position and set upright (the sign has pre-punched holes and can be attached to the white hanging ties at the front of the Malaise trap).
- The trap **MUST** be deployed for the two week period of **September 19 – September 30, 2016** inclusive. If there are any questions or concerns regarding deployment, please contact the School Malaise Trap Program Team at info@malaiseprogram.ca.

Specimen Collection

- Each trap will be accompanied by two specimen collection bottles (500mL plastic Nalgene bottles) that are ready for use. These bottles will be pre-labeled and filled with a preservative, which is 95% ethanol (please note this is a hazardous substance). Ethanol evaporates rapidly so please ensure that the collection bottles are tightly sealed when not attached to the trap. If

there is excessive evaporation or a spill **DO NOT** add anything to the bottle contents. Please contact the School Malaise Trap Program Team immediately at info@malaiseprogram.ca and we will advise on how to proceed.

- Collection bottle one is to be secured to the trap on the **first day of the deployment period: Monday, September 19, 2016.** Remove week one's bottle **at the end of the day on Friday, September 23, 2016.** Collection bottle one, now filled with specimens, should be stored in a secure, cool, and dark location until it and all other Malaise materials are returned back to CBG at the end of the deployment period. **No insect collecting will take place over the weekend of September 24/25, 2016.** Collection bottle two is to be secured to the trap on **Monday, September 26, 2016.** Remove week two's bottle at the **end of the day on Friday, September 30, 2016**; the last day of the deployment period.

Monitoring

- Teachers are asked to visit the trap at least every other day throughout the deployment period to ensure it is standing, secure, and to monitor the catch volume. Teachers are asked to report an estimate of catch volume at the conclusion of the first sampling week (i.e. **Report on Friday, September 23, 2016**). Tick lines on the collection bottles will assist with this reporting. Should there be too few specimens to register via the first tick line, a rough count of the number of specimens is requested. Reporting should be directed to info@malaiseprogram.ca. This email address can also be used to contact the project team at CBG if any issues or concerns arise during the deployment period.
- Teachers are asked to keep a record of scientifically significant events (i.e. date, weather information, catch volumes observed, and incidence of trap disturbance) on the spreadsheet provided. This record should be submitted along with the specimen collection bottles at the end of the collection period. As a suggestion, these records sheets may be hung up in the classroom as a tool for student engagement throughout the deployment period. See document entitled *Collection Records Spreadsheet*.

Submitting Specimens and Results

- At the end of the deployment period, the collection bottles containing the specimens and all other materials are to be couriered back to CBG (refer to the checklist provided at the beginning of this document to ensure all materials are returned). **This package must be dispatched to and received by CBG during the week of October 3 – 7, 2016. If your package is not received by CBG during this week, your school's samples will be excluded from the program (some exceptions may apply based on the location of the school).**
- Please use the original packaging (box, bubble wrap) to ship your specimens etc. back to CBG.
- FedEx will be used to courier your parcel. A pre-filled FedEx waybill has been supplied to you. **Please ensure that the information on your waybill is correct**. Once your package is ready to be shipped, please affix this waybill to the outside of your package and call FedEx (1.800.GoFedEx) to arrange a pick-up. If you are having trouble arranging a pickup, please contact CBG.
- Specimens from the School Malaise Trap Program will be processed at CBG and the results will be delivered to teachers in December via email.
