# Supplementary material for: The School Malaise Trap Program: Coupling educational outreach with scientific discovery
Source: PLoS Biol. 2017 Apr 24;15(4):e2001829. doi: 10.1371/journal.pbio.2001829 (PMC5402927; doi:10.1371/journal.pbio.2001829)
Supplement: S2 Text — (PDF) [file pbio.2001829.s003.pdf]

School Malaise trap Program

# Program Overview: Elementary Schools

## Trap Deployment: September 19 – September 30, 2016

## Project Description

Since 2013, the School Malaise Trap Program has teamed up with thousands of students and educators across Canada in order to explore the insect diversity found in their schoolyards. The program's goal is to encourage students to explore, question and understand the world around them – starting with their own schoolyard. Since its inception, the School Malaise Trap Program has reached over 300 schools, 15,000 students and has collected and sorted over 322,000 insect specimens which were caught in the schoolyards of the participating classes.

Much more impressive still are the scientific findings associated with the School Malaise Trap Program. So far, the School Malaise Trap Program team has sequenced over 85,000 insect specimens representing over 8,000 individual species! Additionally, over 1,200 of the individual species collected were new to the Barcode of Life Data Systems. This means that we were able to assign DNA barcodes and reference data to these species so that they can be easily identified and accessed by experts from around the world.

In addition to receiving the Malaise trap, each school will be provided with engaging material that will allow students to explore the life of a field biologist. Furthermore, through inquiry based learning, students and teachers will have the opportunity to gain insights into Canadian biodiversity and DNA barcoding. The goal of the project is to assess the number of species (insect biodiversity) in each schoolyard and to compare diversity among schools, as well as between schools and nearby National Parks and Conservation Areas, when possible.

## DNA Barcoding

DNA barcoding is a genetic approach to species identification. It has been used as a tool for species identification in a wide array of practical applications that include monitoring pests and species at risk, surveillance of invasive and endangered species, and the authentication of food and herbal products.

The Centre for Biodiversity Genomics (CBG) at the Biodiversity Institute of Ontario (BIO), University of Guelph, is the birthplace of DNA barcoding and home of the world's largest facility for the production and analysis of DNA barcodes. Although DNA barcoding was developed in Canada, it has yet to find its way into classrooms in this country despite its relevance to curricula at many levels. We hope to address this gap with the continuation of the School Malaise Trap Program.

## Participating Schools

Dozens of classes from public, catholic, and independent schools from across Canada are participating in this year's program. A full list of the participating test sites will be published, along with their collection data, at the end of the program.

## Elementary School Programming

(Please note that details of this program may change)

The School Malaise Trap Program relates to several grades and topics within the elementary science curriculum across all Canadian provinces. Specifically, our program will assist teachers in addressing the following teaching expectations and standards:

Explore science and technology vocabulary, including (but not limited to): community, population, habitat, food chain, biodiversity, organism, vertebrate, invertebrate, classification, morphology, adaptation, DNA

Analyse the positive and negative impacts of human interactions with natural habitats and communities

Demonstrate an understanding of why habitats have limits to the number of organisms they can support

Demonstrate an understanding of a community as a group of interacting species sharing a habitat

Classify arthropods (i.e. fly, bee/wasp, beetle, moth/butterfly, true bugs, ticks/mites, etc.)

Identify and describe distinguishing characteristics of different groups of arthropods

Investigate the arthropods found in different habitats and understand similarities and differences

Understand and follow safety procedures for outdoor activities and field work, including working with soils and natural materials

### The Elementary School Program is to be broken down into the following 4 core components:

### 1. Classroom

Each classroom will be provided with engaging material which will introduce students and teachers to the concept of biodiversity and DNA barcoding.

Specific instructions will be provided regarding the use and set-up of the Malaise Trap.

### 2. Malaise Trap Deplo yment

Each teacher will deploy a Malaise Trap in their schoolyard (alternate locations for deployment can be discussed) for a scheduled 2 week period at the end of September 2016 (September 19 – September 30).

Traps are to be monitored for disturbance and specimen accumulation throughout the duration of deployment.

The timing of deployment must be respected. Consistency across all Malaise Traps at all of the participating schools will result in accurate data comparisons and allow timely delivery of the results.

At the end of the 2 week period, teachers will take down the trap and return all materials to CBG by FedEx (waybill provided). Once specimens arrive at CBG, they will be analyzed and barcoded.

### 3. Results

In December, trap results will be emailed to teachers.

### 4. Supplementary Material

Direction to additional resources will be provided to teachers who wish to implement additional lessons/activities to complement the School Malaise Trap Program.

Supplementary teaching resources will be available online for quick and easy access (malaiseprogram.ca).

## Resources

School Malaise Trap Program: malaiseprogram.ca

The BIObus Blog: BIObus.ca

International Barcode of Life: iBOL.org

The Centre for Biodiversity Genomics: biodiversitygenomics.net

Biodiversity Institute of Ontario: biodiversity.ca

DNA Barcoding Blog: dna-barcoding.blogspot.ca
