# Supplementary material for: The School Malaise Trap Program: Coupling educational outreach with scientific discovery
Source: PLoS Biol. 2017 Apr 24;15(4):e2001829. doi: 10.1371/journal.pbio.2001829 (PMC5402927; doi:10.1371/journal.pbio.2001829)
Supplement: S4 Text — (PDF) [file pbio.2001829.s005.pdf]

It's a Malaise Trap!

What is a Malaise Trap?
This tent-like device is an effective collecting tool used for scientific research, particularly to carry out biodiversity inventories.

How does it work?
When insects encounter the black mesh panel, most will naturally go up towards the white coloured roof in an attempt to escape. There they encounter a collection bottle and become permanently trapped. The collection bottle is filled with ethanol to preserve the organisms and their DNA. The trap can be deployed indefinitely, with only the bottle replaced periodically (usually once a week), making it a very low maintenance and low cost sampling technique.

Why is there a Malaise Trap here?
A Malaise Trap is generally placed in a natural flyway where it can collect hundreds of flying insects over time, thereby providing a very detailed understanding of local biodiversity. The collected data can be utilized for bio-monitoring programs that seek to track both long-term trends as well as to evaluate the impacts of human activities on species diversity.

What happens next?
The collection bottle from the trap is brought back from the field, and each specimen is processed and barcoded at the Canadian Centre for DNA barcoding (CCDB). This DNA barcode and other information about the specimen is added to the Barcode of Life Data Systems (BOLD), an online workbench that provides tools for the management and analysis of DNA barcode data.

What is a DNA Barcode?
DNA barcodes are unique genetic signatures that can be used just like the barcodes on consumer products, but to rapidly and accurately identify species. Barcoding requires a tiny piece of tissue, e.g. a bug leg, because DNA is found in every cell of every organism. This technique allows us to identify species from all life stages (such as eggs and larvae) and in all forms (such as processed foods and partial remains).

Why identify species?
Earth is home to many millions of species, but 250 years of traditional study has described fewer than 2 million of them. The ability to identify a species has many practical applications in our daily life, such as detecting market substitution in seafood, adulterations of meat products, monitoring the health of ecosystems, and tracking the spread of disease vectors in the face of global climate change.

Where can I learn more?
- Canadian National Parks Malaise Program - biobus.ca
- The School Malaise Trap Program - malaiseprogram.ca
- International Barcode of Life Project - ibol.org
- Canadian Centre for DNA Barcoding - ccdb.ca
- Barcode of Life Data Systems - boldsystems.org
- DNA Barcode Blog - dna-barcoding.blogspot.ca

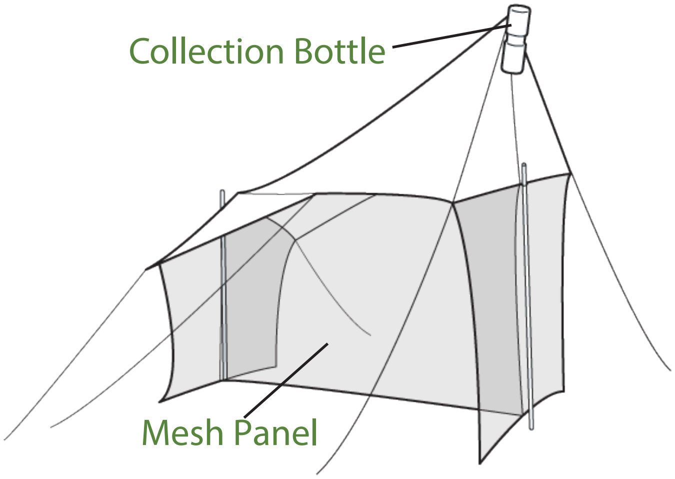

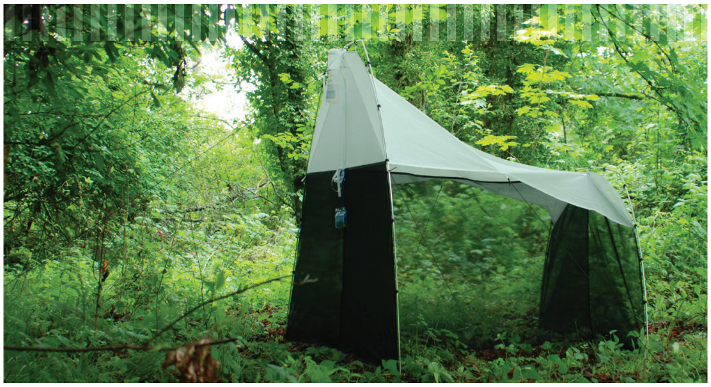

Monitoring the Environment

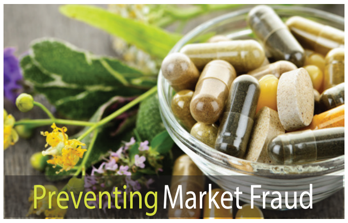

Preventing Market Fraud

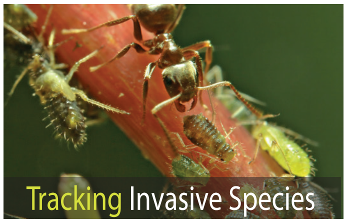

Tracking Invasive Species

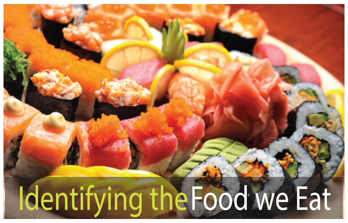

Identifying the Food we Eat

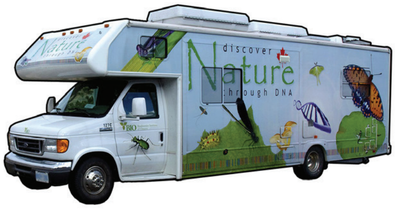

@Smtp_Canada

BIO
