# Supplementary material for: The School Malaise Trap Program: Coupling educational outreach with scientific discovery
Source: PLoS Biol. 2017 Apr 24;15(4):e2001829. doi: 10.1371/journal.pbio.2001829 (PMC5402927; doi:10.1371/journal.pbio.2001829)
Supplement: S5 Text — (PDF) [file pbio.2001829.s006.pdf]

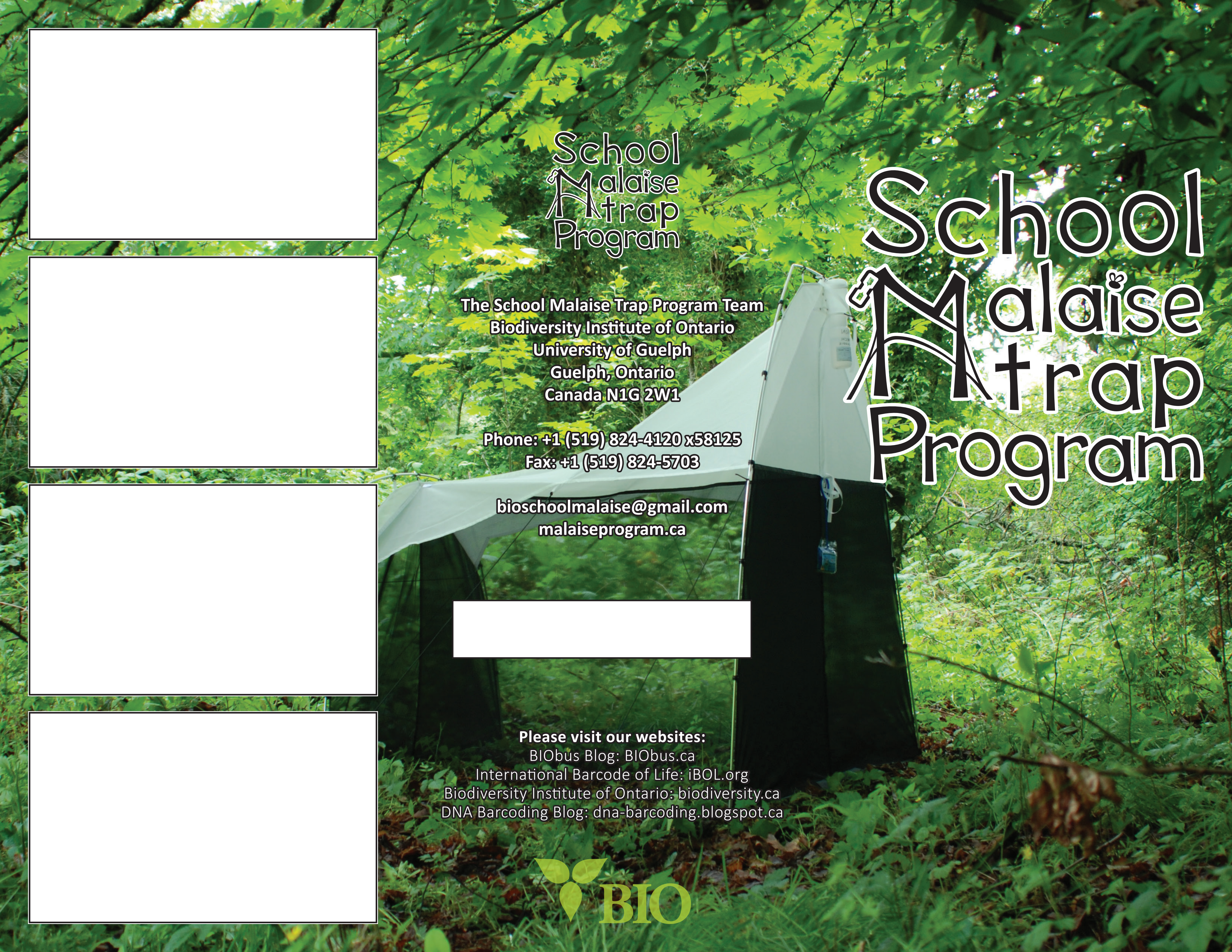

School Malaise Trap Program

School Malaise Trap Program

The School Malaise Trap Program Team
Biodiversity Institute of Ontario
University of Guelph
Guelph, Ontario
Canada N1G 2W1

Phone: +1 (519) 824-4120 x58125
Fax: +1 (519) 824-5703

bioschoolmalaise@gmail.com
malaiseprogram.ca

Please visit our websites:
BIObus Blog: BIObus.ca
International Barcode of Life: iBOL.org
Biodiversity Institute of Ontario: biodiversity.ca
DNA Barcoding Blog: dna-barcoding.blogspot.ca

BIO

School Malaise trap Program

The Program

Since 2011, the Biodiversity Institute of Ontario (BIO) has teamed up with multiple schools and thousands of students across Canada to explore the insect diversity in their schoolyards through DNA barcoding, a genetic technique for identifying organisms. Using a Malaise trap, a small tent-like apparatus, each school has collected hundreds of insect specimens.

The School Malaise Trap Program's goal is to allow students a sneak peak into the life of biologists working with DNA barcoding. Comprehensive lesson plans accompany the project and address multiple specific expectations across the Canadian elementary and secondary curriculums.

Once each class has set up their Malaise trap for two weeks during the fall/spring, the samples will be analyzed at BIO and each class will receive a report summarizing the insects collected at each school. It will highlight new discoveries, and whenever possible will make comparisons between schools and nearby National Parks and Conservation Areas.

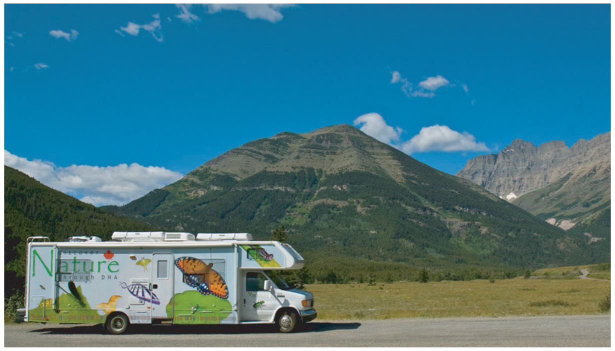

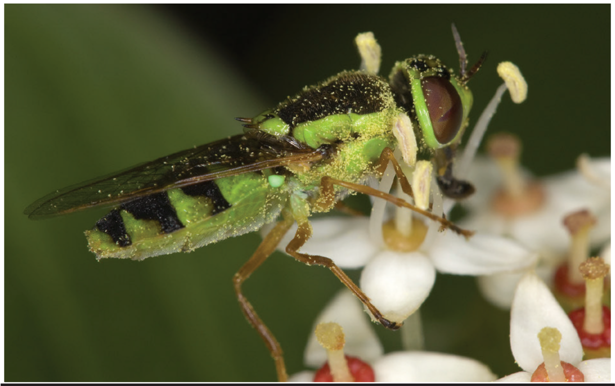

Why Collect Insects?

The Malaise Trap is a very effective tool used for scientific research, particularly to carry out biodiversity inventories. This project is part of an ongoing effort to collect insects in order to document the small and often overlooked cohabitants of our planet.

Insects perform many vital roles in our ecosystem. Burrowing bugs such as ants and beetles dig tunnels that provide channels for water and air, benefiting plants. Bees play a major role in pollinating fruit trees and flower blossoms. Gardeners love to see insect predators because they control certain harmful insect populations, such as aphids and caterpillars. Finally, many insects eat dead and decaying materials, thereby reintroducing nutrients into the soil.

What is a DNA Barcode?

DNA barcodes are unique genetic signatures that can be used just like the barcodes on consumer products, but to identify species. DNA barcoding requires only a tiny piece of tissue, because DNA is found in every cell of every organism. This technique allows us to identify species from all life stages (such as eggs and larvae) and in all forms (such as processed foods and partial remains).

DNA barcoding was invented at the Biodiversity Institute of Ontario in 2003 and the technique has spread quickly around the planet. Through the School Malaise Trap Program and the various educational activities that the program provides, DNA barcoding is finding its way into classrooms in Canada, demonstrating its relevance to the curriculum at many levels.

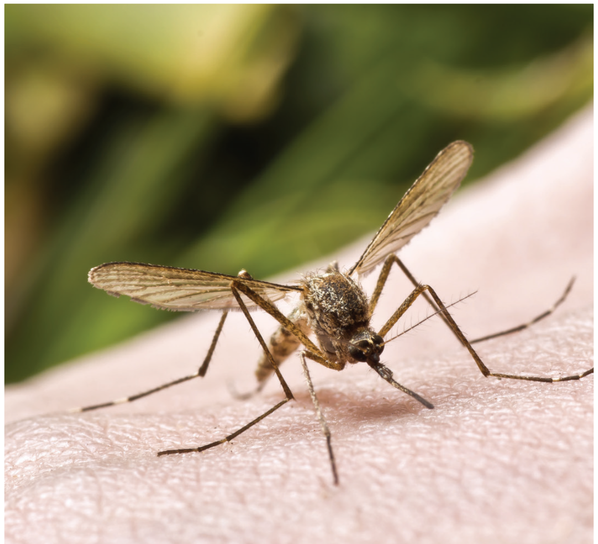

Common House Mosquito (*Culex pipiens*)
