# Supplementary material for: The School Malaise Trap Program: Coupling educational outreach with scientific discovery
Source: PLoS Biol. 2017 Apr 24;15(4):e2001829. doi: 10.1371/journal.pbio.2001829 (PMC5402927; doi:10.1371/journal.pbio.2001829)
Supplement: S1 Document Collection — (ZIP) [file pbio.2001829.s007.zip › Activity 1 - Bug Sort.docx]

Purpose:

- To understand the advantages of grouping or classifying of organisms on the basis of common characteristics, and the necessity of a common classification scheme.

Strategy Overview:

Students will explore different classification schemes by grouping arthropod shapes

Assessment Strategies:

- Observation of group work

Prior Knowledge and Skills:

- Understanding of basic groups of animals such as arthropods (in particular arachnids and insects)

Suggested Timing:

- 45 minutes

Materials:

- Scissors
- Printout of **InsectBLM.pdf** for each student

Instructions:

Each student should cut out all bug shapes from the bug sheet and sort them into groups based on characteristics they have in common. Students may use fairly specific characteristics such as wings, legs, or antenna, or they may use more general grouping such as insects, spiders, etc., or they may use some combination of each. Students can then attempt to sort those using different characteristics, and come up with an entirely different classification scheme. As they compare their schemes with others in their class, they will see that their classification schemes will not be the only way to classify organisms. The focus in this activity should be on the fact that classification schemes are human inventions; there is no right or wrong classification scheme.

This can lead to a discussion around the necessity for a common classification scheme in order to for scientists all over the world to communicate using the same language and terminology. There are approximately 1.7 million species of living things, with the possibility of millions more to be discovered. This raises questions about how we can simplify the study and collection of information of so many species.

These discussions encourage students to appreciate the role of science in their understanding of the world.

Extensions:

- Students could use a dichotomous key to identify the insect order of most of the silhouettes in **InsectBLM.pdf.** A basic key can be found at the [AMNH resource pages](http://www.amnh.org/learn/biodiversity_counts/ident_help/Text_Keys/arthropod_keyA.htm).

Additional Information:

[Animal Diversity Web](http://animaldiversity.ummz.umich.edu/) (accessed April 12, 2013)

Animal Diversity Web is a site provided by the University of Michigan’s Museum of Zoology. It is full of pictures, specimens, information and classification for hundreds of different animals. The classification charts show how each animal is classified and provides links to more information on each category of classification.

[The 5 Kingdoms of Life](http://www.davidlnelson.md/Cazadero/FiveKingdoms.htm) (accessed April 12, 2013)

A site to learn more about Carl Linneaus and his system of binomial nomenclature and about why there are 5 different kingdoms.

[Kids.Net.Au - Scientific Classification](http://encyclopedia.kids.net.au/page/sc/Scientific_classification) (accessed April 2013)

Formatted like an encyclopedia entry, this site contains information on the history of classification, mnemonics to help remember the different categories, clues for remembering the naming suffixes, examples of specific animal classifications and links to more information.

[Encyclopedia of Life](http://eol.org/) (accessed April 2013)

A growing resource to increase awareness and understanding of living nature through an Encyclopedia of Life that gathers, generates, and shares knowledge in an open, freely accessible and trusted digital resource.
