# Supplementary material for: The School Malaise Trap Program: Coupling educational outreach with scientific discovery
Source: PLoS Biol. 2017 Apr 24;15(4):e2001829. doi: 10.1371/journal.pbio.2001829 (PMC5402927; doi:10.1371/journal.pbio.2001829)
Supplement: S1 Document Collection — (ZIP) [file pbio.2001829.s007.zip › Activity 1 - Bug Sort BLM.docx]

Classification helps us to impose order and a general plan on the diversity of living things. Scientists have always tried to organize and classify the objects, including living organisms, around them. Classification can be defined as grouping organisms according to similarities. This means that organisms that share similar features are placed in one group.

With artificial classification you can use any grouping you like.

You could put all the animals that fly in the same group. This group would then include birds, bats and many insects. You could put all animals that live in water and have streamlined, fish-like bodies in the same group. This group would then include fish and whales. Artificial classification systems are also used as the basis for keys that biologists use to identify organisms.

In this activity you will learn how to develop an artificial classification that works for an assemblage of bugs.

1. Each student should cut out all bug shapes from the bug sheet.
2. Sort the shapes into groups based on characteristics they have in common. Pick the ones that you think are the most reasonable.
3. Compare your classification system with the one your neighbour used.
4. Together try come up with a totally different classification scheme
5. Discuss in your class why it is important to have a common classification scheme

Example for a simple scientific key to identify species.
