# Supplementary figures and images for: The School Malaise Trap Program: Coupling educational outreach with scientific discovery
Source: PLoS Biol. 2017 Apr 24;15(4):e2001829. doi: 10.1371/journal.pbio.2001829 (PMC5402927; doi:10.1371/journal.pbio.2001829)

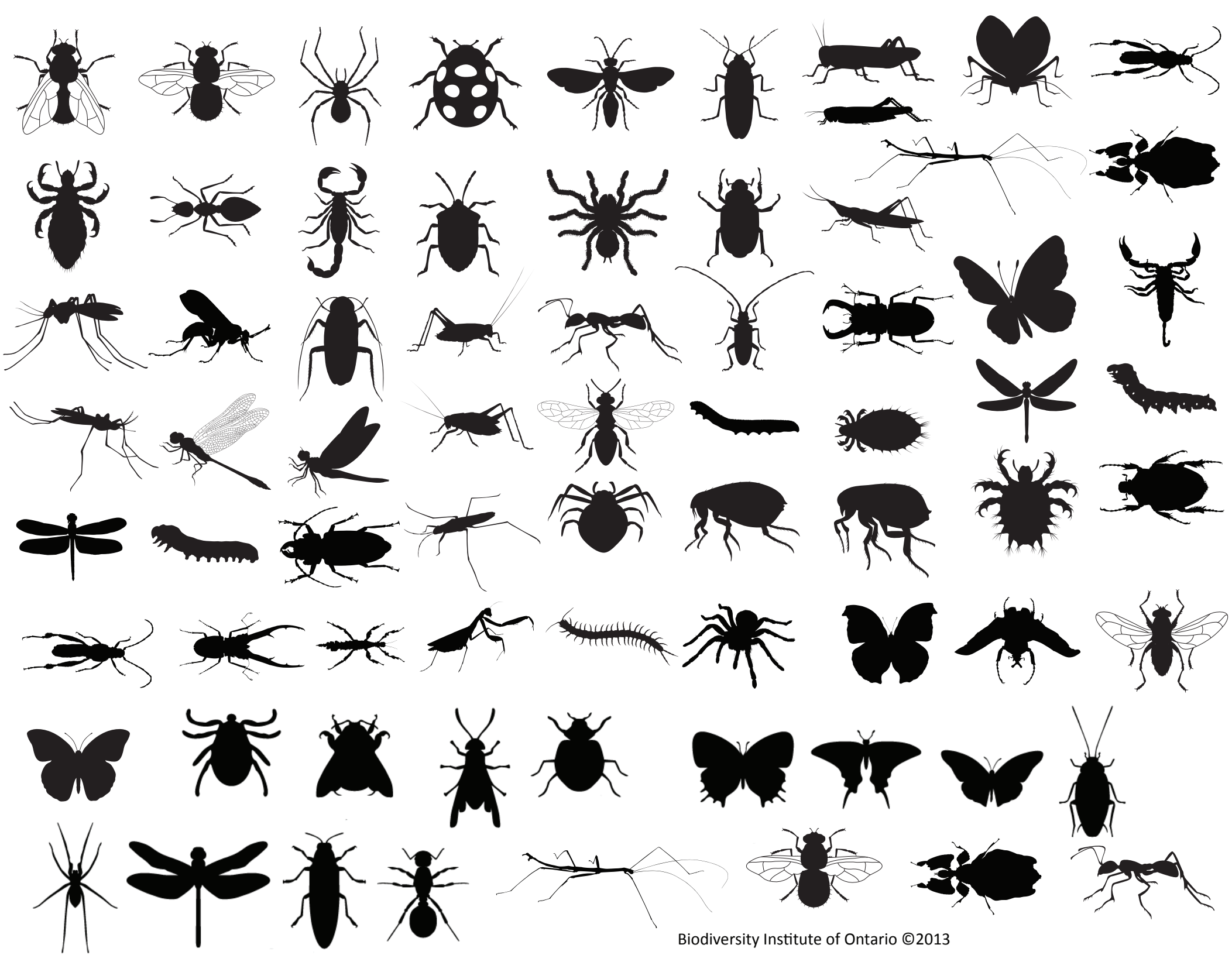

Supplement: S1 Document Collection — (ZIP) [file pbio.2001829.s007.zip › InsectBLM.pdf]
