# Supplementary material for: The School Malaise Trap Program: Coupling educational outreach with scientific discovery
Source: PLoS Biol. 2017 Apr 24;15(4):e2001829. doi: 10.1371/journal.pbio.2001829 (PMC5402927; doi:10.1371/journal.pbio.2001829)
Supplement: S2 Document Collection — (ZIP) [file pbio.2001829.s008.zip › Strawberry DNA Extraction Backgrounder.docx]

In recent years, it is not uncommon to read articles on DNA in both scientific and popular magazines. DNA is regularly mentioned in the news and is often featured in TV detective or crime-scene investigation dramas. DNA, also known as Deoxyribonucleic Acid, is a long molecule that holds the genetic information for all living beings, be it plant, animal or simple microorganisms. It is capable of copying itself and can synthesize RNA (Ribonucleic Acid). In more evolved or complex forms of life, DNA is contained in the nucleus of the cells. Except for the red blood cells of mammals, which are devoid of a nucleus, all cells of a living being have their own DNA. The cells of an organism use certain parts of the DNA molecule, or genes, to produce the proteins they need to function.

The procedure of this activity exploits the fact that the external membrane of cells and that of their nuclei are composed of lipid walls that can be broken down using a simple detergent. The first operation in this procedure is to break-up the fruit into a pulp or mush so that the cells are separated from each other as much as possible thereby exposing them to the detergent. The detergent is added to the fruit pulp to release the DNA encapsulated by the lipid membranes. The salt neutralizes the negative charges on the DNA and thus enables the DNA strands to stick together. It also causes proteins and carbohydrates to precipitate.

It is necessary to filter the mixture to separate the nucleic acid from the remains of the cellular membranes. Finally, the DNA is precipitated in alcohol where it becomes visible. The DNA you obtain using this procedure can be observed with a microscope and can be used for other experiments like electrophoresis.
