# Supplementary material for: The School Malaise Trap Program: Coupling educational outreach with scientific discovery
Source: PLoS Biol. 2017 Apr 24;15(4):e2001829. doi: 10.1371/journal.pbio.2001829 (PMC5402927; doi:10.1371/journal.pbio.2001829)
Supplement: S2 Document Collection — (ZIP) [file pbio.2001829.s008.zip › Strawberry DNA Extraction Worksheet.docx]

**Group members: _______________________________________ Date: ___________________ Page 1/2**

In this exercise you will be extracting DNA from strawberries.

*Your group will need:*

***Tools***

- Safety goggles – 1 per student
- 1 sandwich-sized resalable bag
- Measuring cup
- Teaspoon and tablespoon
- Glass or small bowl
- Cheesecloth
- Funnel
- Tall drinking glass
- Test tube or small glass jar
- Skewer

***Materials***

- 3 strawberries (green tops removed)
- ½ teaspoon salt
- 1/3 cup water
- 1 tablespoon liquid dishwashing detergent
- Isopropyl (rubbing) alcohol

*Procedure*

1. Chill the rubbing alcohol in the freezer. (You'll need it later.)
2. Each group member should put on a pair of safety goggles.
3. Mix the salt, water, and dishwashing detergent in a glass or small bowl. Set the mixture aside. This is your extraction liquid.
4. Line the funnel with the cheesecloth, and put the funnel's tube into the glass.
5. Put the strawberries in the plastic bag and push out all the extra air. Seal it tightly.
6. With your fingers, squeeze and smash the strawberry mixture for 2 minutes.
7. Add 3 tablespoons of the extraction liquid you made in Step 3 to the strawberries in the bag. Push out all the extra air and reseal the bag.
8. Squeeze the strawberry mixture with your fingers for 1 minute.
9. Pour the strawberry mixture from the bag into the funnel. Let it drip into the glass until there is no liquid left in the funnel.
10. Throw away the cheesecloth and the strawberry pulp inside. Pour the contents of the glass (filtrate) into the test tube or small glass jar so it is 1/4 full.

**Page 2/2**

1. Tilt the test tube or jar and **very slowly** pour the cold rubbing alcohol down the side. The alcohol should form a thin layer on top of the strawberry liquid. (Don't let the alcohol and strawberry liquid mix. The DNA collects between the two layers!)
2. Dip the skewer into the test tube where the alcohol and strawberry layers meet. Pull up the skewer. The whitish, stringy stuff is DNA containing strawberry genes!

*Key Terms:*

- **DNA**: Stands for Deoxyribonucleic acid. This is the hereditary material in cells that contains the instructions for producing the cell and enabling it to function.
- **Extraction**: A procedure to obtain a substance by chemical or mechanical action.
- **Filtrate**: The material collected after it passes through a filter.

***Questions:***

1. What is the role of the detergent?
2. What is the role of the salt?
3. What is the role of the alcohol?
4. Do you think you could extract DNA if you were to use a fruit or vegetable other than strawberries? Explain.
