# Supplementary material for: The School Malaise Trap Program: Coupling educational outreach with scientific discovery
Source: PLoS Biol. 2017 Apr 24;15(4):e2001829. doi: 10.1371/journal.pbio.2001829 (PMC5402927; doi:10.1371/journal.pbio.2001829)
Supplement: S2 Document Collection — (ZIP) [file pbio.2001829.s008.zip › Strawberry DNA Extraction Activity Description.docx]

# **ACTIVITY 2: Strawberry DNA Extraction**

**MINDS-ON 3: Page 1 of 2**

Purpose:

- To understand the process used to extract DNA from cells
- To correctly and accurately follow a scientific protocol

Strategy Overview:

Students will perform a strawberry DNA extraction laboratory activity in order to gain a basic understanding of how DNA can be extracted from tissues and cells.

Assessment Strategies:

- Observation of group work during the laboratory activity
- Completion of questions on worksheet

Prior Knowledge and Skills:

- Understanding of cellular structure (i.e., cell and nuclear membranes, DNA, proteins)

Suggested Timing:

- 45 minutes

Materials:

- **Strawberry DNA Extraction Answer Page** (For teacher use)

**For each small group (2-3 students)**

- **Strawberry DNA Extraction Worksheet** – 1 copy per small group
- **Strawberry DNA Extraction Backgrounder** – 1 copy per small group

***Tools***

- Safety goggles – 1 per student
- 1 sandwich-sized resealable bag
- Measuring cup
- Teaspoon and tablespoon
- Glass or small bowl
- Cheesecloth
- Funnel
- Tall drinking glass
- Test tube or small glass jar
- Skewer

***Materials***

- 3 strawberries (green tops removed)
- ½ teaspoon salt
- 1/3 cup water
- 1 tablespoon liquid dishwashing detergent
- Isopropyl (rubbing) alcohol

Instructions:

**MINDS-ON 3: Page 2 of 2**

- Review the instructions for the activity with the students. **Note: ensure that students follow the warnings on the side of the bottle of isopropyl alcohol – in particular, it should be used in a well-ventilated area. It is flammable and should be kept away from open flames. It is also poisonous, so should not be ingested or otherwise consumed.**
- Hand out materials to each group. Have students follow the instructions on the **Strawberry DNA Extraction Worksheet**. Walk around as students complete the extraction, answering questions and guiding as necessary.
- Students can use the **Strawberry DNA Extraction Backgrounder** or do other research in order to answer the questions on the second page.

Extensions:

- Students can place a small amount of the extracted DNA on a dry microscopic slide. They should not attempt to make a wet slide as the DNA will go into solution and will not be visible. Have the students observe the DNA at various levels of magnification and make notes and drawings.
- DNA can also be obtained from other types of fruit and vegetables, such as bananas, spinach, kiwi, raspberries, onions, and even oatmeal. Different groups of students could try different fruits and vegetables.

Additional Information:

Resources related to DNA extraction

- [DNA Extraction: Biotechniques Virtual Lab](http://learn.genetics.utah.edu/content/labs/extraction/) (Accessed Apr. 12, 2013)
