# Supplementary material for: The School Malaise Trap Program: Coupling educational outreach with scientific discovery
Source: PLoS Biol. 2017 Apr 24;15(4):e2001829. doi: 10.1371/journal.pbio.2001829 (PMC5402927; doi:10.1371/journal.pbio.2001829)
Supplement: S2 Document Collection — (ZIP) [file pbio.2001829.s008.zip › Strawberry DNA Extraction Answer Page.docx]

1. What is the role of the detergent?

*Detergent acts to pull apart the phospholipids and proteins that make up the membranes surrounding the cell and nucleus. Once these membranes are broken apart, the DNA is released from the cell.*

2. What is the role of the salt?

*The salt neutralizes the negative charges on the DNA and thus enables the DNA strands to stick together. It also causes proteins, carbohydrates, and other cell debris to precipitate.*

3. What is the role of the alcohol?

*The DNA released from the cell nucleus is dissolved in the water/detergent/salt/strawberry solution and cannot be seen. DNA precipitates out of solution in alcohol, where it can be seen.*

4. Do you think that you could extract DNA if you were to use a fruit or vegetable other than strawberries? Explain.

*Since DNA is in the cells of every living organism, this technique could be used to extract DNA from any fruit or vegetable.*
