# Supplementary material for: The School Malaise Trap Program: Coupling educational outreach with scientific discovery
Source: PLoS Biol. 2017 Apr 24;15(4):e2001829. doi: 10.1371/journal.pbio.2001829 (PMC5402927; doi:10.1371/journal.pbio.2001829)
Supplement: S3 Document Collection — (ZIP) [file pbio.2001829.s009.zip › Activity 1 - Bug ID Activity Description.docx]

Bug ID: Activity Description

Purpose:

- To use the Barcode of Life Data Systems (BOLD) to identify DNA samples

Strategy Overview:

Students will be introduced to the Barcode of Life Data Systems (BOLD) which is a valuable resource for researchers from around the world. BOLD is an online tool for collecting, managing and analyzing DNA barcodes. Students will become familiar with BOLD by using a few of its many features to determine the species identity of some sample DNA sequences.

Assessment Strategies:

- Observation of online work (digital literacy) and class discussion
- Completion of **Bug ID Worksheet**

Prior Knowledge and Skills:

- Familiarity with DNA barcoding (such as from classroom talks)
- Basic understanding of Linnaean classification system from previous grades
- Ability to use the internet for research (as students will need to match scientific names with common names)
- Familiarity with binomial nomenclature (two part species name, also known as Latin name)

Suggested Timing:

- 45 minutes (30 minutes for internet searching, 15 minute for discussion)
- The internet activity could be done as a homework assignment

Materials (Please access online at malaiseprogram.ca/for-teachers/resources/)

- **Bug ID Worksheet** – 1 copy per student (Note: Students will need access to this digitally as they will need to copy and paste long sequences of letters into BOLD)
- **Bug ID Answer Page** (for teacher use)
- Students will need access to the internet to complete this activity

Instructions:

-
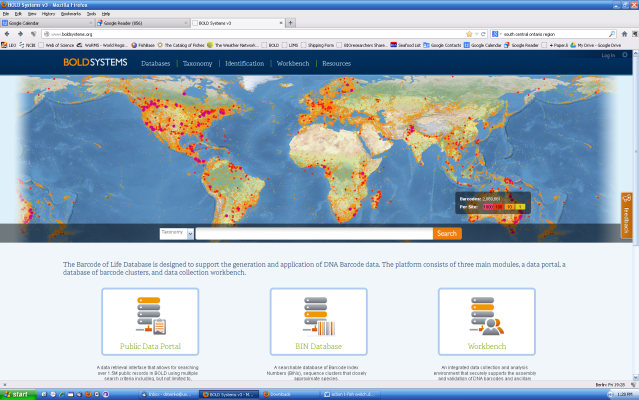
Students may complete this activity independently or in pairs and they will need access to the internet.
- The students will need to go to <http://www.boldsystems.org/> where they will use the Identification engine for this activity.
-
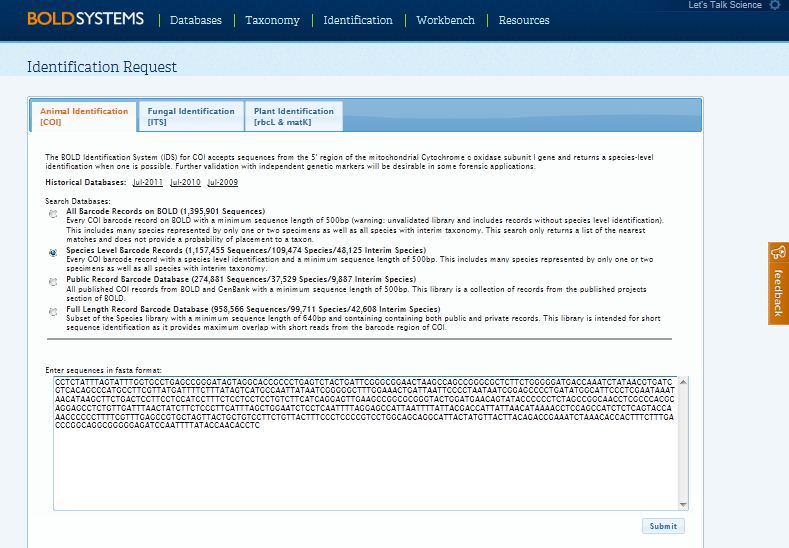
Once on the page, they will need to click on **Identification** in the menu bar at the top of the page. They should see a page which has the **Animal Identification (COI)** tab selected and the **Species Level Barcode Records** button selected.

**ACTION 1: Page 2 of 4**

- Using a digital copy (MSWord or PDF) of the **Bug ID Worksheet**, students will copy and paste the first DNA sequence from the chart into the box on the page where it says ***Enter sequences in Fasta format:*** and then click **submit** at the bottom right side of the screen.

**FASTA format** is a text-based format for representing nucleotide sequences. The nucleotides are represented using single-letter codes (A, C, T and G).


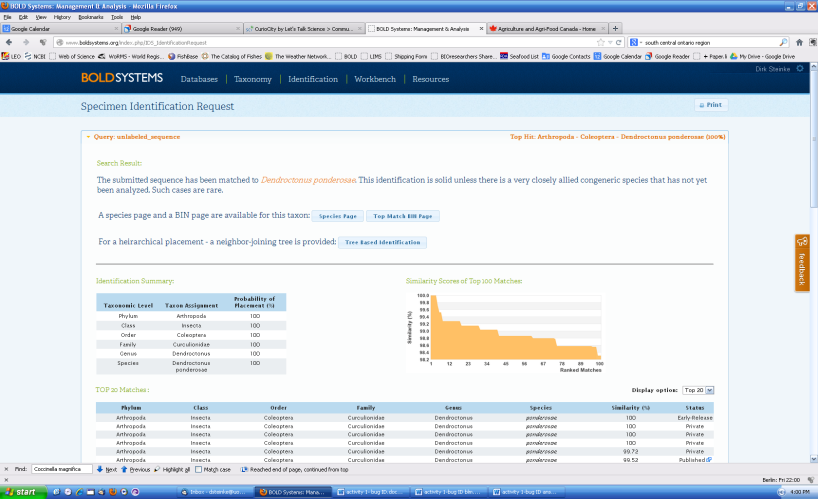


- The search results (Top Hit) will show up on the right and will let you know the genus and species of the animal in the sample. The page will also provide a link to a species page with more information including images and a map where individuals of the species have been encountered.
- Students will need to repeat this process for each DNA sequence and then fill in the rest of the chart.


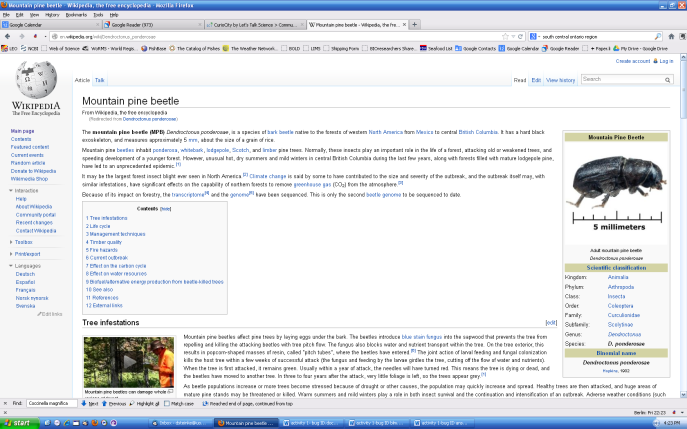


- Students will need to go to other internet sources to
  determine the common name of the species found
  in BOLD and if it is native to Canada.
-
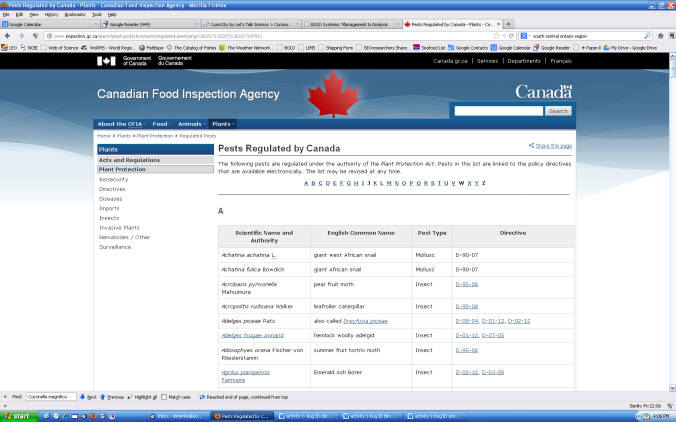
They will also use the [CFIA page](http://www.inspection.gc.ca/plants/plant-protection/pests/regulated-pests/eng/1363317115207/1363317187811) to determine if a species is considered a pest in Canada.
- The student **Bug ID Worksheet** can be checked for accuracy using the **Bug ID Answer Page**.
- As a class, discuss the results. Information about what insect pest species are and what damage they can cause can be found e.g. on the [Canadian Forest Service](http://www.nrcan.gc.ca/forests/insects-diseases/13361) page.

**ACTION 1: Page 3 of 4**

- Questions for discussion can include:
  - *Should potential pests be treated the same wherever they occur?*
  - *Is there a way we can now define when an organism is and is not a pest?*

Extensions:

- Students can explore other tools on the BOLD website. For example, if you click on Taxonomy in the top menu, you can choose an animal and follow its taxonomy. At each level (phylum, class, order, family, genus, species), you can see statistics such as the number of specimen records, the number of species with barcodes and where the samples were collected, as well as which countries the specimens were collected from. If you go down to the species level, you can click on Access Published & Released Data for the species. After clicking an entry, you can see the COI sequence for the species.
- Provide students with species names and have them determine if the given species has a barcode using the Taxonomy tree (see above).
- If you click on Databases in the top menu and then choose Public Data Portal, you can put your location into the search engine and see if any species have been catalogued in your area.

Additional Information:

**Barcoding Resources**

- [DNA barcode to identify intercepted gypsy moths](http://ibol.org/wp-content/uploads/2011/06/DNA-barcode-to-identify-intercepted-gypsy-moths-_-NSW-Department-of-Primary-Industries.pdf) (Accessed Mar. 29, 2013)

This article from Agriculture Today (2011) describes how DNA barcoding can help to distinguish between pest and

non-pest gypsy moth species in Australia.

- [Barcode of Life: Guelph-based DNA database a digital Noah’s ark aiming to ID all living organisms](http://news.nationalpost.com/2013/03/29/international-barcode-of-life/) (Accessed Mar. 29, 2013)

This article from The *National Post* (2013) describes barcoding and the International Barcode of Life Project (iBOL).

**ACTION 1: Page 4 of 4**

- [New Tool for Tracking a Voracious Pest](http://www.ars.usda.gov/is/pr/2012/120508.htm) (Accessed Mar. 29, 2013)

An article in *Agricultural Research* (2012) about DNA barcoding and the identification of the Russian wheat aphid.
