# Supplementary material for: The School Malaise Trap Program: Coupling educational outreach with scientific discovery
Source: PLoS Biol. 2017 Apr 24;15(4):e2001829. doi: 10.1371/journal.pbio.2001829 (PMC5402927; doi:10.1371/journal.pbio.2001829)
Supplement: S3 Document Collection — (ZIP) [file pbio.2001829.s009.zip › Activity 1 - Bug ID Answer Page.doc]

| **Sample Name** | **DNA Sequence (COI base pair sequence)** | **Top Hit in BOLD (Genus and species)** | **Common name of this animal** | **Is it a native species?**  **Y/N** | **Pest**  **Species? Y/N** |
| --- | --- | --- | --- | --- | --- |
| 1 | AATAATATATTTTATTTTTGCCATATGATCAGGAATAATTGGATCATCTATAAGATTATTAATTCGAATAGAACTTAGACATCCTGGAATATGAATTAATAATGATCAAATTTATAATTCTTTAGTAACAAGTCATGCATTTTTAATAATTTTTTTTATAGTTATACCATTTATAATTGGAGGATTTGGAAATTACCTAATTCCTTTAATATTAGGATCTCCTGATATAGCTTTTCCACGAATAAATAATATTAGATTTTGATTACTTCCACCATCTCTTCTAATACTTCTTCTAAGAAATTTATTTACTCCAAACGTTGGAACTGGATGAACTGTTTATCCTCCTTTATCATCATATTTATTTCATTCATCACCTTCTGTTGATATTGCAATTTTTTCATTACATATAACCGGAATTTCTTCAATTATTGGATCTTTAAATTTTATTGTAACTATTATATTAATAAAAAATTTTTCATTAAATTATGATCAAATTAACTTATTTTCTTGGTCAGTTTGTATTACAGTAATATTATTAATTTTATCTTTACCAGTTTTAGCAGGAGCAATTACTATACTCCTTTTTGATCGAAATTTTAATACATCTTTCTTTGATCCAATAGGAGGAGGTGATCCTATTCTTTATCAACACTTATTT | *Bombus perplexus* | Perplexing bumble bee OR Confusing bumble bee | Y | N |
| 2 | ATTTATCGCTTATTATCGGCCACTTTATCGAATAAATGATTATTTTCTTCTAATCATAAAGATATTGGAACATTATATTTCTTATTCGGAATATGAGCCGGAATAATTGGGACCTCTTTAAGAATTTTAATTCGTCTTGAATTAGGAACTACTAATAGATTAATTGGAAATGACCAAATTTATAATGTAATTGTAACAGCTCATGCCTTCATTATAATTTTTTTTATAGTTATACCGATTATAATTGGAGGATTTGGAAATTGACTTGTTCCTTTAATAATTGGAGCACCTGATATAGCTTTCCCTCGATTAAATAATATAAGATTTTGACTACTCCCACCTGCCTTAACCTTACTTATTATTAGAAGATTAGTGGAAATAGGTGCAGGAACTGGATGAACTGTCTATCCTCCTTATCCTCTAACTTAGCTCATAATGGGCCTTCAGTAGATTTAGTAATTTTTAGTTTACACTTAGCAGGTATCTCATCTATTTTAGGAGCCGTAAATTTTATTTCACTATTATAAATATACGACCATTTGGCATAAA | *Coccinella septempunctata* | Seven-spotted lady beetle OR Seven-spot ladybird | N | N |
| 3 | CACACTCTACTTTATTTTTGGGGCCTGATCAGGAATAGTAGGAACAGCCCTTAGACTACTAATTCGAGCAGAATTAGGAAATCCAGGAGCATTAATTGGCAATGACCAAATCTATAATGTTATTGTTACTGCTCATGCTTTTATTATAATTTTCTTCATGGTTATACCTATTATAATAGGGGGATTTGGAAACTGATTGGTTCCTTTAATGTTAGGGGCACCTGATATAGCTTTCCCCCGAATAAATAATATAAGATTCTGGCTACTCCCTCCCTCTTTAACATTACTTTTAATAAGAAGAATAGTCGAAAGAGGAGCAGGGACTGGTTGGACAGTATATCCACCATTAGCGGCTAATATTGCCCATAGAGGGGGCTCTGTTGACTTAGCAATTTTTAGACTTCATTTAGCTGGAATCTCCTCAATTCTAGGGGCAATTAATTTTATTACAACAGTAATTAATATGCGAGCAATAGGAATAACCTTAGACCAAACACCTCTACTAGTCTGATCTATTGCTATTACAGCCCTTCTTCTTCTTTTATCCTTACCTGTTCTTGCGGGAGCAATTACCATACTTTTAACAGATCGAAACTTAAATACATCATTCTTTGACCCGGCTGGAGGAGGAGACCCTATTCTTTATCAACATCTCTTT | *Agrilus planipennis* | Emerald ash borer | N | Y |
| 4 | ATTCTATATTTTATCTTTGCTATTTGATCAGGAATAATTGGCTCCTCTATAAGAATAATCATTCGACTAGAGTTAGGATCTCCTGATTCATTAATTCTTAATGATCAAACTTTTAATACCATCGTTACAAGTCATGCTTTTATTATAATTTTTTTTATAGTTATACCTTTTATAATCGGAGGATTTGGTAATTTTCTAATTCCACTTATACTAGGATCTCCTGATATGGCTTACCCCCGTTTAAATAATATAAGATTTTGATTACTTCCCCCATCGATCTCCTTGTTAATCTTAAGAAATTTTATTAATGAAGGATCTGGAACTGGTTGAACTATCTACCCCCCTCTATCATCAAATACCTTTCATAGTGGCCCCTCCATTGACCTAACTATCTTTTCTCTCCATATTGCCGGTATATCCTCAATTATAGGAGCAATCAATTTTATTTCAACAATTATAAATATACATAATTCCAATATTTCCTTAGATAAAATCCCCTTGTTAGTATGATCCATCCTTATTACAGCTATTCTCCTTCTTCTGTCCCTACCTGTTCTAGCAGGA | *Camponotus modoc* | Western carpenter ant | Y | Y |
| 5 | TACTTTATATTTTATTTTTGGAATTTGAGCAGGAATAGTTGGGACATCTTTAAGTCTTTTAATTCGAACAGAATTAGGAACTCCTGGATCTTTAATTGGTGATGATCAAATTTATAATACTATTGTTACAGCTCATGCTTTTATTATAATTTTTTTTATAGTTATACCAATTATAATTGGAGGATTTGGTAATTGATTAGTACCTCTAATATTAGGAGCTCCTGATATAGCTTTCCCCCGAATAAATAATATAAGATTTTGACTTTTACCCCCATCATTAATTTTATTAATTTCAAGAAGAATCGTAGAAAATGGTGCAGGAACAGGATGAACAGTTTACCCCCCACTTTCATCAAATATTGCTCATAGAGGATCTTCTGTAGATCTAGCTATTTTTTCTTTACATTTAGCTGGAATTTCATCTATTTTAGGAGCTATTAATTTTATTACTACAATCTTAAATATACGAATTAATAATATAACATTTGATCAAATACCTTTATTTGTTTGAGCAGTAGGTATTACAGCTCTTCTTTTATTACTTTCTTTACCAGTTTTAGCAGGAGCAATTACTATACTTCTTACTGATCGAAATTTAAATACTTCTTTTTTTGATCCTGCTGGTGGAGGAGACCCTATTTTATATCAACATTTATTT | *Danaus plexippus* | Monarch butterfly | Y | N |
| 6 | ACATTATATTTTATTTTTGGAATTTGAGCTGGAATAGTAGGAACATCACTAAGAATTATTATTCGAATGGAATTAAGAGGCATTAATAATTTTATTGGTAATGATCAAATTTATAATGTAATCGTAACTGCTCATGCATTTATTATAATTTTTTTTATAGTTATGCCTATTTTAATTGGTGGATTTGGTAACTGATTAATTCCTATCATATTAGGAGCACCAGATATAGCATTTCCACGAATAAATAATTTAAGATTTTGATTTTTGCCCCCTTCTCTTATATTATTATTAATTAGAAGATTAGTTGAAATAGGAACCGGAACAGGATGAACAGTATATCCTCCTTTGTCTTCATCAATTGCCCATACTGGCTCTTCTGTTGATTTTTCAATTTTTTCATTACATATTGCTGGTATTTCTTCTATTTTAGGAGCTATTAATTTTATTTCAACAATATTAAATATAAAAATTAAATTTATTAAATTTGATCAAATCTCACTTTTTACATGATCTGTTTTAATTACTGCTGTACTTCTTCTTTTATCTTTACCAGTTCTTGCTGGAGCTATTACAATATTATTAACTGAT | *Mayetiola destructor* | Barley midge | N | Y |
| 7 | AACATTATATTTTATTTTTGGAATTTGAGCAGGAATAGTAGGAACTTCATTAAGATTACTAATTCGAGCAGAATTAGGAACCCCCGGATCTTTAATTGGAGATGATCAAATTTATAATACAATTGTAACAGCTCATGCATTTATTATAATTTTTTTTATAGTAATACCTATTATAATTGGAGGATTTGGTAATTGATTAGTTCCTTTAATATTAGGAGCTCCTGATATAGCATTCCCACGTATAAATAATATAAGATTTTGACTTTTACCCCCTTCTTTAACATTATTAATTTCTAGAAGTATTGTTGAAAATGGAGCTGGAACTGGATGAACAGTTTACCCACCTTTATCATCTAATATTGCTCATAGAGGCAGATCTGTAGATTTAGCTATTTTTTCTCTTCATTTAGCTGGAATTTCATCAATTATAGGAGCAGTTAATTTTATTACAACAATTATTAATATACGAATTAACAGCTTATCTTTTGATCAAATACCATTATTTGTTTGAGCTGTTGGAATTACAGCATTTTTATTACTTTTATCTTTACCTGTATTAGCAGGAGCTATTACTATACTATTAACTGATCGAAATTTAAATACATCATTTTTTGACCCAGCTGGAGGAGGAGATCCTATTTTATATCAACATTTATTT | *Macroglossum stellatarum* | Humming-bird Hawkmoth | Y | N |
| 8 | ACATTATATTTTATTTTTGGTTTATGATCAGGACTAATTGGAACATCTTTAAGATTAATTATTCGAGCTGAATTAGGAAATCCAGGATCATTAATTGGAGATGATCAAATCTATAATACTATTGTTACAGCTCATGCTTTTATCATAATTTTTTTTATAGTTATACCTATTATAATTGGAGGATTTGGAAATTGATTAGTACCTTTAATGCTTGGAGCTCCTGATATAGCTTTCCCCCGAATAAATAATATAAGATTCTGACTATTACCCCCATCTTTAACTTTATTAATTTCTAGAAGTATTGTAGAAAATGGAGCAGGAACAGGTTGAACAGTATACCCTCCATTATCATCTAATATTGCCCATAGAGGAAGATCAGTAGATTTAGCTATTTTTTCCCTTCATTTAGCTGGAATTTCATCTATCTTAGGAGCAATTAATTTTATTACAACAATTATTAATATACGATCTAATGGAATATCTTTTGATCGAATACCTTTATTTGTTTGAGCTGTAGGTATTACTGCTTTATTATTACTTTTATCTTTACCTGTTTTAGCAGGTGCTATTACTATATTATTAACTGATCGAAATTTAAATACATCCTTTTTTGAT | *Acrolepiopsis assectella* | Leek moth OR Onion leaf miner | N | Y |
| 9 | TACTTTATATTTTATTTTTGGAGCTTGGTCAGGAATAGTAGGAACATCACTAAGACTTATTATTCGCACAGAACTAGGTATCCCAGGAAGTTTAATTGGAGATGATCAAATTTATAATACTATTGTAACTGCCCACGCATTTATTATAATTTTTTTTATAGTTATACCAATTATAATTGGGGGATTCGGAAACTGATTAATTCCTTTAATGTTAGGAGCACCTGATATAGCCTTTCCACGATTAAATAATATAAGATTCTGATTATTACCTCCTTCATTAACATTTTTGCTTCTTAGAAGAATTATTGATAAAGGGGCAGGAACTGGATGAACAGTATATCCTCCTTTAGCTTCTAATATTTCCCATGAAGGATCCTCAGTTGACTGTGCTATTTTCAGGCTTCATATAGCAGGAGTATCCTCTATCCTTGGAGCTATTAATTTTATTTCTACAATTATAAATATAGCCCCTTCAGGAATAAAATTAGATCGTTTAACGTTATTTACATGAGCAGTAAAAATTACAGCTATCTTATTATTGTTATCATTACCAGTATTAGCTGGAGCCATCACTATACTTTTAACAGACCGAAATATCAATACTACTTTTTTTGACCCCTCCGGTGGAGGAGATCCTATTCTCTATCAACACTTATTT | *Dendroctonus ponderosae* | Mountain pine beetle | Y | Y |
