# Supplementary material for: The School Malaise Trap Program: Coupling educational outreach with scientific discovery
Source: PLoS Biol. 2017 Apr 24;15(4):e2001829. doi: 10.1371/journal.pbio.2001829 (PMC5402927; doi:10.1371/journal.pbio.2001829)
Supplement: S3 Document Collection — (ZIP) [file pbio.2001829.s009.zip › Activity 1 - Bug ID Worksheet.doc]

Page 1/4

A pest is a plant or animal detrimental to humans or human concerns such as agriculture or livestock production. Often animals are considered pests because they cause damage to agriculture by feeding on crops or parasitising livestock. Using the method below and the species DNA samples on the following pages, you will determine if some of the species collected are actually considered pests in Canada.

1. Go to [http://www.boldsystems.org](http://www.boldsystems.org/)
2. Click on **Identification** in the menu bar at the top of the page. Make sure that page that comes up has the **Animal Identification (COI)** tab selected and the **Species Level Barcode Records** button selected.
3. Copy and paste in the DNA sequence from the chart on the following pages into the box on the page and then click **submit** at the bottom right side of the screen. The identification engine may take a few minutes to complete the search.

**FASTA format** is a text-based format for representing nucleotide sequences. The nucleotides are represented using single-letter codes (A, C, T and G).

4. The search results (**Top Hit**) will show up on the right and will let you know the genus and species of the animal in the sample. The page will also provide a link to a species page with more information including image and a map where individuals of the species have been encountered.
5. Go back one page or click on **Identification** in the menu bar at the top of the page again to enter the next DNA sequence.
6. Complete the rest of the chart. You will need to use the internet to determine the common name of the species found in BOLD and if it is native to Canada.
7. Use the [CFIA page](http://www.inspection.gc.ca/plants/plant-protection/pests/regulated-pests/eng/1363317115207/1363317187811) to determine if a species is considered a pest in Canada.

Name: _____________________________________	Due Date: _________________________	Page 2/4

| **Sample Name** | **DNA Sequence (COI base pair sequence)** | **Top Hit in BOLD (Genus and species)** | **Common name of this animal** | **Is it a native species?**  **Y/N** | **Pest**  **Species? Y/N** |
| --- | --- | --- | --- | --- | --- |
| 1 | AATAATATATTTTATTTTTGCCATATGATCAGGAATAATTGGATCATCTATAAGATTATTAATTCGAATAGAACTTAGACATCCTGGAATATGAATTAATAATGATCAAATTTATAATTCTTTAGTAACAAGTCATGCATTTTTAATAATTTTTTTTATAGTTATACCATTTATAATTGGAGGATTTGGAAATTACCTAATTCCTTTAATATTAGGATCTCCTGATATAGCTTTTCCACGAATAAATAATATTAGATTTTGATTACTTCCACCATCTCTTCTAATACTTCTTCTAAGAAATTTATTTACTCCAAACGTTGGAACTGGATGAACTGTTTATCCTCCTTTATCATCATATTTATTTCATTCATCACCTTCTGTTGATATTGCAATTTTTTCATTACATATAACCGGAATTTCTTCAATTATTGGATCTTTAAATTTTATTGTAACTATTATATTAATAAAAAATTTTTCATTAAATTATGATCAAATTAACTTATTTTCTTGGTCAGTTTGTATTACAGTAATATTATTAATTTTATCTTTACCAGTTTTAGCAGGAGCAATTACTATACTCCTTTTTGATCGAAATTTTAATACATCTTTCTTTGATCCAATAGGAGGAGGTGATCCTATTCTTTATCAACACTTATTT |  |  |  |  |
| 2 | ATTTATCGCTTATTATCGGCCACTTTATCGAATAAATGATTATTTTCTTCTAATCATAAAGATATTGGAACATTATATTTCTTATTCGGAATATGAGCCGGAATAATTGGGACCTCTTTAAGAATTTTAATTCGTCTTGAATTAGGAACTACTAATAGATTAATTGGAAATGACCAAATTTATAATGTAATTGTAACAGCTCATGCCTTCATTATAATTTTTTTTATAGTTATACCGATTATAATTGGAGGATTTGGAAATTGACTTGTTCCTTTAATAATTGGAGCACCTGATATAGCTTTCCCTCGATTAAATAATATAAGATTTTGACTACTCCCACCTGCCTTAACCTTACTTATTATTAGAAGATTAGTGGAAATAGGTGCAGGAACTGGATGAACTGTCTATCCTCCTTATCCTCTAACTTAGCTCATAATGGGCCTTCAGTAGATTTAGTAATTTTTAGTTTACACTTAGCAGGTATCTCATCTATTTTAGGAGCCGTAAATTTTATTTCACTATTATAAATATACGACCATTTGGCATAAA |  |  |  |  |
| 3 | CACACTCTACTTTATTTTTGGGGCCTGATCAGGAATAGTAGGAACAGCCCTTAGACTACTAATTCGAGCAGAATTAGGAAATCCAGGAGCATTAATTGGCAATGACCAAATCTATAATGTTATTGTTACTGCTCATGCTTTTATTATAATTTTCTTCATGGTTATACCTATTATAATAGGGGGATTTGGAAACTGATTGGTTCCTTTAATGTTAGGGGCACCTGATATAGCTTTCCCCCGAATAAATAATATAAGATTCTGGCTACTCCCTCCCTCTTTAACATTACTTTTAATAAGAAGAATAGTCGAAAGAGGAGCAGGGACTGGTTGGACAGTATATCCACCATTAGCGGCTAATATTGCCCATAGAGGGGGCTCTGTTGACTTAGCAATTTTTAGACTTCATTTAGCTGGAATCTCCTCAATTCTAGGGGCAATTAATTTTATTACAACAGTAATTAATATGCGAGCAATAGGAATAACCTTAGACCAAACACCTCTACTAGTCTGATCTATTGCTATTACAGCCCTTCTTCTTCTTTTATCCTTACCTGTTCTTGCGGGAGCAATTACCATACTTTTAACAGATCGAAACTTAAATACATCATTCTTTGACCCGGCTGGAGGAGGAGACCCTATTCTTTATCAACATCTCTTT |  |  |  |  |

Name: _____________________________________	Due Date: _________________________	Page 3/4

| **Sample Name** | **DNA Sequence (COI base pair sequence)** | **Top Hit in BOLD (Genus and species)** | **Common name of this animal** | **Is it a native species?**  **Y/N** | **Pest**  **Species? Y/N** |
| --- | --- | --- | --- | --- | --- |
| 4 | ATTCTATATTTTATCTTTGCTATTTGATCAGGAATAATTGGCTCCTCTATAAGAATAATCATTCGACTAGAGTTAGGATCTCCTGATTCATTAATTCTTAATGATCAAACTTTTAATACCATCGTTACAAGTCATGCTTTTATTATAATTTTTTTTATAGTTATACCTTTTATAATCGGAGGATTTGGTAATTTTCTAATTCCACTTATACTAGGATCTCCTGATATGGCTTACCCCCGTTTAAATAATATAAGATTTTGATTACTTCCCCCATCGATCTCCTTGTTAATCTTAAGAAATTTTATTAATGAAGGATCTGGAACTGGTTGAACTATCTACCCCCCTCTATCATCAAATACCTTTCATAGTGGCCCCTCCATTGACCTAACTATCTTTTCTCTCCATATTGCCGGTATATCCTCAATTATAGGAGCAATCAATTTTATTTCAACAATTATAAATATACATAATTCCAATATTTCCTTAGATAAAATCCCCTTGTTAGTATGATCCATCCTTATTACAGCTATTCTCCTTCTTCTGTCCCTACCTGTTCTAGCAGGA |  |  |  |  |
| 5 | TACTTTATATTTTATTTTTGGAATTTGAGCAGGAATAGTTGGGACATCTTTAAGTCTTTTAATTCGAACAGAATTAGGAACTCCTGGATCTTTAATTGGTGATGATCAAATTTATAATACTATTGTTACAGCTCATGCTTTTATTATAATTTTTTTTATAGTTATACCAATTATAATTGGAGGATTTGGTAATTGATTAGTACCTCTAATATTAGGAGCTCCTGATATAGCTTTCCCCCGAATAAATAATATAAGATTTTGACTTTTACCCCCATCATTAATTTTATTAATTTCAAGAAGAATCGTAGAAAATGGTGCAGGAACAGGATGAACAGTTTACCCCCCACTTTCATCAAATATTGCTCATAGAGGATCTTCTGTAGATCTAGCTATTTTTTCTTTACATTTAGCTGGAATTTCATCTATTTTAGGAGCTATTAATTTTATTACTACAATCTTAAATATACGAATTAATAATATAACATTTGATCAAATACCTTTATTTGTTTGAGCAGTAGGTATTACAGCTCTTCTTTTATTACTTTCTTTACCAGTTTTAGCAGGAGCAATTACTATACTTCTTACTGATCGAAATTTAAATACTTCTTTTTTTGATCCTGCTGGTGGAGGAGACCCTATTTTATATCAACATTTATTT |  |  |  |  |
| 6 | ACATTATATTTTATTTTTGGAATTTGAGCTGGAATAGTAGGAACATCACTAAGAATTATTATTCGAATGGAATTAAGAGGCATTAATAATTTTATTGGTAATGATCAAATTTATAATGTAATCGTAACTGCTCATGCATTTATTATAATTTTTTTTATAGTTATGCCTATTTTAATTGGTGGATTTGGTAACTGATTAATTCCTATCATATTAGGAGCACCAGATATAGCATTTCCACGAATAAATAATTTAAGATTTTGATTTTTGCCCCCTTCTCTTATATTATTATTAATTAGAAGATTAGTTGAAATAGGAACCGGAACAGGATGAACAGTATATCCTCCTTTGTCTTCATCAATTGCCCATACTGGCTCTTCTGTTGATTTTTCAATTTTTTCATTACATATTGCTGGTATTTCTTCTATTTTAGGAGCTATTAATTTTATTTCAACAATATTAAATATAAAAATTAAATTTATTAAATTTGATCAAATCTCACTTTTTACATGATCTGTTTTAATTACTGCTGTACTTCTTCTTTTATCTTTACCAGTTCTTGCTGGAGCTATTACAATATTATTAACTGAT |  |  |  |  |

Name: _____________________________________	Due Date: _________________________	Page 4/4

| **Sample Name** | **DNA Sequence (COI base pair sequence)** | **Top Hit in BOLD (Genus and species)** | **Common name of this animal** | **Is it a native species?**  **Y/N** | **Pest**  **Species? Y/N** |
| --- | --- | --- | --- | --- | --- |
| 7 | AACATTATATTTTATTTTTGGAATTTGAGCAGGAATAGTAGGAACTTCATTAAGATTACTAATTCGAGCAGAATTAGGAACCCCCGGATCTTTAATTGGAGATGATCAAATTTATAATACAATTGTAACAGCTCATGCATTTATTATAATTTTTTTTATAGTAATACCTATTATAATTGGAGGATTTGGTAATTGATTAGTTCCTTTAATATTAGGAGCTCCTGATATAGCATTCCCACGTATAAATAATATAAGATTTTGACTTTTACCCCCTTCTTTAACATTATTAATTTCTAGAAGTATTGTTGAAAATGGAGCTGGAACTGGATGAACAGTTTACCCACCTTTATCATCTAATATTGCTCATAGAGGCAGATCTGTAGATTTAGCTATTTTTTCTCTTCATTTAGCTGGAATTTCATCAATTATAGGAGCAGTTAATTTTATTACAACAATTATTAATATACGAATTAACAGCTTATCTTTTGATCAAATACCATTATTTGTTTGAGCTGTTGGAATTACAGCATTTTTATTACTTTTATCTTTACCTGTATTAGCAGGAGCTATTACTATACTATTAACTGATCGAAATTTAAATACATCATTTTTTGACCCAGCTGGAGGAGGAGATCCTATTTTATATCAACATTTATTT |  |  |  |  |
| 8 | ACATTATATTTTATTTTTGGTTTATGATCAGGACTAATTGGAACATCTTTAAGATTAATTATTCGAGCTGAATTAGGAAATCCAGGATCATTAATTGGAGATGATCAAATCTATAATACTATTGTTACAGCTCATGCTTTTATCATAATTTTTTTTATAGTTATACCTATTATAATTGGAGGATTTGGAAATTGATTAGTACCTTTAATGCTTGGAGCTCCTGATATAGCTTTCCCCCGAATAAATAATATAAGATTCTGACTATTACCCCCATCTTTAACTTTATTAATTTCTAGAAGTATTGTAGAAAATGGAGCAGGAACAGGTTGAACAGTATACCCTCCATTATCATCTAATATTGCCCATAGAGGAAGATCAGTAGATTTAGCTATTTTTTCCCTTCATTTAGCTGGAATTTCATCTATCTTAGGAGCAATTAATTTTATTACAACAATTATTAATATACGATCTAATGGAATATCTTTTGATCGAATACCTTTATTTGTTTGAGCTGTAGGTATTACTGCTTTATTATTACTTTTATCTTTACCTGTTTTAGCAGGTGCTATTACTATATTATTAACTGATCGAAATTTAAATACATCCTTTTTTGAT |  |  |  |  |
| 9 | TACTTTATATTTTATTTTTGGAGCTTGGTCAGGAATAGTAGGAACATCACTAAGACTTATTATTCGCACAGAACTAGGTATCCCAGGAAGTTTAATTGGAGATGATCAAATTTATAATACTATTGTAACTGCCCACGCATTTATTATAATTTTTTTTATAGTTATACCAATTATAATTGGGGGATTCGGAAACTGATTAATTCCTTTAATGTTAGGAGCACCTGATATAGCCTTTCCACGATTAAATAATATAAGATTCTGATTATTACCTCCTTCATTAACATTTTTGCTTCTTAGAAGAATTATTGATAAAGGGGCAGGAACTGGATGAACAGTATATCCTCCTTTAGCTTCTAATATTTCCCATGAAGGATCCTCAGTTGACTGTGCTATTTTCAGGCTTCATATAGCAGGAGTATCCTCTATCCTTGGAGCTATTAATTTTATTTCTACAATTATAAATATAGCCCCTTCAGGAATAAAATTAGATCGTTTAACGTTATTTACATGAGCAGTAAAAATTACAGCTATCTTATTATTGTTATCATTACCAGTATTAGCTGGAGCCATCACTATACTTTTAACAGACCGAAATATCAATACTACTTTTTTTGACCCCTCCGGTGGAGGAGATCCTATTCTCTATCAACACTTATTT |  |  |  |  |
